# Association of incident dialysis modality with patient survival: a systematic review and meta-analysis

**DOI:** 10.1186/s12882-025-04530-4

**Published:** 2025-10-24

**Authors:** Luca Nardelli, Matteo Passerini, Dalia Zubidat, Anna Sikharulidze, Elisa Cicero, Carlo Alfieri, Manuel Podestà, Giuseppe Castellano, Mohammad Hassan Murad

**Affiliations:** 1 Division of Nephrology, Dialysis and Kidney Transplantation, Fondazione IRCCS Ca’ Granda Ospedale Maggiore Policlinico, Via della Commenda 15, 20122 Milan, Italy; 2 Department of Clinical Sciences and Community Health, Università degli studi di Milano, Milan, Italy; 3 Department of Pathophysiology and Transplantation, Università degli studi di Milano, Milan, Italy; 4 Infectious Diseases Unit, Luigi Sacco Hospital, ASST Fatebenefratelli Sacco, Milan, Italy; 5 Department of Internal Medicine, Montefiore Hospital, New Rochelle (NY), USA; 6 Division of Public Health, Infectious Diseases and Occupational Medicine, Department of Medicine, Mayo Clinic, Rochester, MN USA

**Keywords:** Peritoneal dialysis, Hemodialysis, Mortality, Survival, Diabetes, Systematic review, Metanalysis

## Abstract

**Background:**

Although the choice of dialysis technique is based on several factors, patient survival is undoubtedly one of the most relevant. In a context where randomization to either peritoneal dialysis (PD) or hemodialysis (HD) proved to be extremely challenging, previous meta-analyses were greatly limited due to the inclusion of historical studies.

**Methods:**

We performed a systematic review by searching multiple databases up to April 22nd, 2022. The primary outcome was the association between dialysis modality (PD vs. HD) and mortality assessed via hazard ratios (HR). Subgroup analyses were conducted to explore potential sources of heterogeneity, including sex, age, diabetes, dialysis vintage, geographical location, HD access, and study cohort inclusion period.

**Results:**

Database search yielded 5317 citations, from which, 27 observational studies met the eligibility criteria, including 1 033 362 incident dialysis patients. The pooled mortality HR for PD versus HD was 1.01 (95% CI 0.93–1.10). Heterogeneity was substantial (*I*^*2*^ = 94%) and was largely explained by different baseline features of the included populations. A statistically significant subgroup effect was demonstrated for age (> 65 vs.<65 years; *p* = 0.01), geographical location of the studies (Oceania vs. Europe vs. Asia vs. North America; *p* < 0.01), and HD vascular access (central venous catheter vs. arteriovenous fistula; *p* < 0.01, only one study included).

**Conclusions:**

This meta-analysis suggests that overall PD and incentre HD likely carry equivalent survival benefits. However, differences were detected among subgroups based on age, geographic location, HD access type, but not on sex, diabetes status, dialysis vintage and study cohort inclusion period.

**Clinical trial number:**

Not applicable.

**Supplementary Information:**

The online version contains supplementary material available at 10.1186/s12882-025-04530-4.

## Introduction

Chronic kidney disease (CKD) is a progressive and irreversible condition that ultimately leads to end-stage renal disease (ESRD). Renal replacement therapy (RRT), in the form of chronic dialysis or kidney transplantation, is essential for sustaining life in individuals with ESRD. However, due to the shortage of donor kidneys and the presence of comorbidities that may preclude transplantation, dialysis remains the predominant treatment option for most patients with ESRD [1–4]. In the 1980s, the introduction of peritoneal dialysis (PD) into clinical practice, initially raised the question of which dialysis modality, between PD and hemodialysis (HD), should be preferred [5, 6].

Although multiple factors influence dialysis modality choice, patient survival remains a top priority for both clinicians and patients. The SONG-PD initiative lists mortality among the five core outcomes that should be reported in all PD studies [7]. A multinational study of patients and caregivers from Australia, the United States, and Hong Kong similarly identified survival as a key treatment goal [8]. Hole’ s analysis of dialysis preferences demonstrated that even older patients prioritize treatments that extend survival if independence, treatment location, and frequency are acceptable [9]. Reflecting this consistent emphasis on survival, our meta-analysis focused on all-cause mortality as one of the most universally relevant endpoint for comparing PD and HD in contemporary practice.

Several observational studies have previously compared PD and HD in the ESRD population, but the survival benefit of one modality over the other has not yet been determined since results conflict across studies [10–15].

Moreover, although the results were analyzed after stratification by factors such as age, diabetes status, and dialysis duration, patient subgroups that might derive a greater survival benefit from either PD or HD have not yet been clearly identified.

To date, only two randomized controlled trials attempted to compare mortality risk between the two dialysis modalities. However, the first investigation was halted prematurely due to insufficient enrollment [16], while in the second study the primary outcome was changed to health-related quality of life due to insufficient statistical power [17]. Those results showed the enormous challenge, if not the impossibility, to perform an adequately powered random allocation in this setting.

To overcome this hurdle, few meta-analyses aimed at summarizing the evidence deriving from observational studies have been carried out [18–23]. Nevertheless, all these papers included historical cohorts and were mainly restricted either to elderly populations or individuals with diabetes.

However, in the new millennium the fast spread of automated peritoneal dialysis (APD) in clinical practice, along with the advent of icodextrin [24], promoted a better patient fluid control contributing to solve the problem of fast transport in PD [25–28]. During the same period HD-specific improvements have also occurred with the introduction of biocompatible membranes and the diffusion of on-line high-flux hemodiafiltration [29, 30].

However, over the last two decades, a consistent and substantial reduction in mortality rates for patients starting PD has been reported, although such improvements were not observed for HD patients [31]. This differential change in outcomes mandates a new analysis focused on populations treated with modern dialytic techniques to reflect the current therapeutical standards. Hence, whether HD or PD provides a survival advantage to patients with ESRD still remains an unresolved issue.

## Methods

### Data source and search strategy

A comprehensive search of several databases was performed on April 22nd, 2022. Date limits were applied from 2000 forward. Animal studies were excluded. Databases searched were Ovid MEDLINE(R) 1946 to Present and Epub Ahead of Print, In-Process & Other Non-Indexed Citations and Daily, Ovid Embase 1988+, Ovid Cochrane Central Register of Controlled Trials 1991+, Ovid Cochrane Database of Systematic Reviews 2005+, Web of Science Core Collection via Clarivate Analytics (1975+), and Scopus via Elsevier (1788+). The search strategy was designed and conducted by an experienced librarian with input from the study’s investigators. Controlled vocabulary supplemented with keywords was used to search for studies describing the association of incident dialysis modality with patient survival. The actual strategy listing all search terms used and how they are combined is available in the Supplementary. Citations were managed with the reference management program, Covidence^®^, while data were first collected in Microsoft^®^ Excel v.16.86 and then analyzed through R statistical software [version 4.4.0]^32^.

This systematic review’s protocol was prospectively registered in PROSPERO (No. CRD42022328308). We adhered to the adapted PRISMA guidelines for systematic reviews (checklist in the Supplementary).

### Study selection and data extraction

We included studies which satisfied the following inclusion criteria: (i) comprising adult patients (> 18 years old) with end-stage kidney on dialysis treatment; (ii) comparing any chronic PD treatment (continuous ambulatory PD [CAPD]; automated PD [APD] and its variants: continuous cyclic PD [CCPD], nocturnal intermittent PD [NIPD], intermittent PD [IPD] and tidal PD [TPD]) *versus* any type of HD and its variants (hemofiltration, hemodiafiltration, acid-free biofiltration); (iii) providing data on mortality for the patients included with an intention-to-treat analysis; (iv) including patients who started dialysis after 2000 (in case of studies including patients before and after 2000, we include them if there were enough data to extract only the patients from 2000 onwards); (v) classifying the patients according to dialysis modality received 90 days post-initiation of therapy; and (vi) with at least 50 patients per group. We excluded studies (i) which evaluate combined HD and PD strategies; (ii) where hemodialysis comprises intensive dialysis (i.e. greater than 3.5 times per week, or greater than 6 h per treatment); (iii) where the comparison was between home-HD and PD; (iv) which included the comparison in patients coming back to dialysis after kidney transplant failure; (v) where the results were only presented as as-treated analysis; and (vi) where patients who shifted dialysis method were censored or not included.

Two reviewers (L.N. and D.Z.) screened all titles and abstracts independently. Studies included at this level by either reviewer were included for full-text screening by the same reviewer pair. Discrepancies were solved through discussion or by consultation of a third reviewer (M.P.). Extracted data included study design, year of publication, start and end date of patients’ inclusion, number of patients for each group (HD and PD), and main characteristics of the included patients (sex, age, presence of diabetes, coronary artery disease [CAD], congestive heart failure [CHF], and peripheral vascular disease [PAD]).

### Quality assessment and certainty of evidence

Risk of bias assessment was performed using the Newcastle-Ottawa tool for observational studies [33]. Potential disagreements were resolved through discussion. Certainty of evidence (CoE) of the primary outcome was assessed using the GRADE approach [34].

### Statistical analysis

The primary outcome was mortality as assessed by time-to-event analysis and reported using hazard ratios (HR). Therefore, we collected from each study a pre-computed HR and the corresponding confidence interval (CI) comparing PD and HD. HR’s and their standard errors were pooled using a generic inverse variance approach and a random-effects model with a restricted maximum likelihood ratio estimator of between study heterogeneity. We planned several a priori determined subgroup analyses to explore heterogeneity. These analyses were based on risk of bias, sex, age, diabetes status, dialysis vintage (duration), geographic location (continent of study origin), dialysis access type, and year of the patients’ inclusion in the study. We tested the interaction between subgroups and generated a P value for the difference based on heterogeneity statistic Q [35]. All analyses are performed using R statistical software [32]. Meta-analyses were performed using the R package “meta” and its subgroup analysis function using the “subgroup” argument [36].

## Results

### Search results and characteristics of included studies

**Fig. 1:**
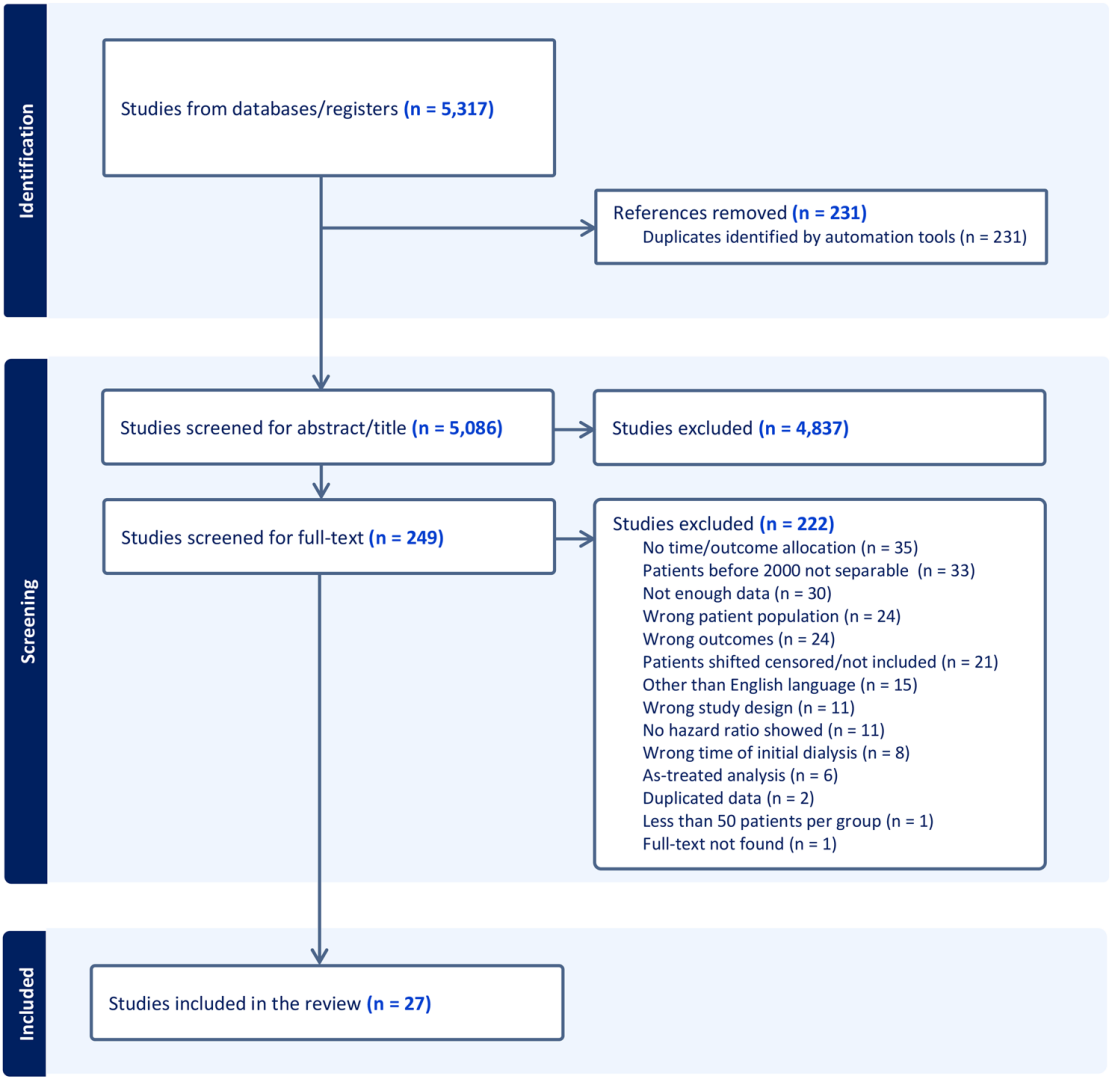
PRISMA flow diagram

The results of the literature search are presented in the PRISMA flow diagram in Fig. 1. The initial search identified 5317 citations. After duplicate removal and exclusion on title and abstract screening, 249 studies qualified for a full text review. Two hundreds and twenty-two studies were excluded for specific reasons (Fig. 1). Consequently, a total of 27 studies met our eligibility criteria contributing to the final meta-analysis [13–15, 37–60]. The characteristics of the included studies are summarized in Table 1.

**Table 1:**
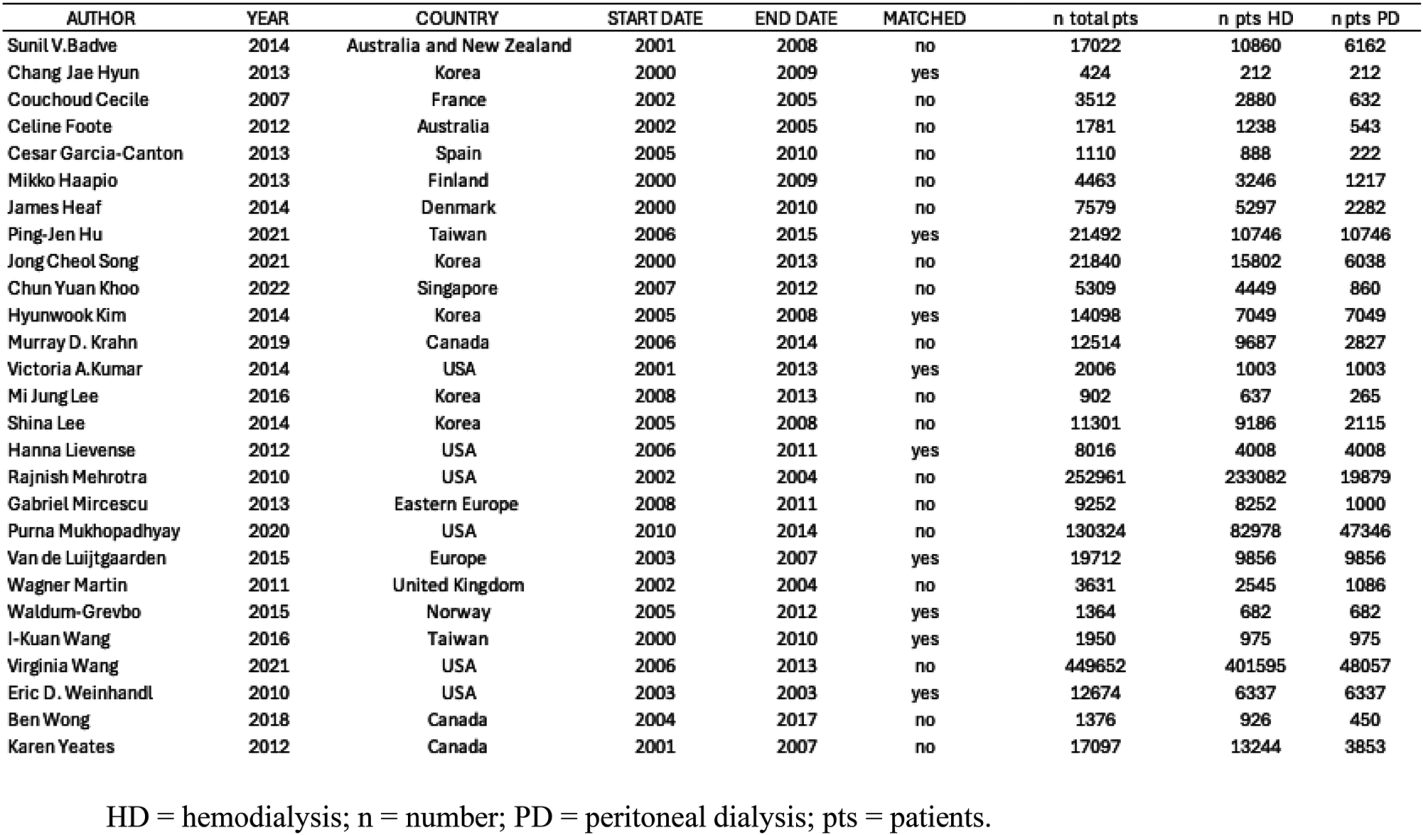
Characteristics of the included studies

All analyzed papers were retrospective cohort studies published between 2007 and 2021: twenty-two studies were registry-based, three were from multi-center cohorts [41, 49, 59] and one single-center study [38]. Cohort periods ranged from 2000 to 2015. There was wide geographical variability, with nine studies conducted on North American populations [13, 14, 47, 48, 51, 53, 58–60], an eight on European cohorts [15, 39, 41, 42, 52, 54–56], an additional eight studies reporting on Asian patients [38, 43, 45, 46, 49, 50, 57, 61], and two on dialysis patients from Australia/New Zealand [37, 40], as shown in Table 1.

Few studies compared mortality risks in populations with specific characteristics, such as those with either diabetes [49], a previous history of stroke [57] or age >75 years [39, 40].

All included studies enrolled incident patients who initiated dialysis between 2000 and 2017. In total, 1,033,362 patients were analyzed, of whom 847,660 started on HD and 185,702 on PD. Among these, 81,736 patients underwent a matching process in the original studies (Table 1). The main characteristics of the included populations are summarized in Table 2.

**Table 2:**
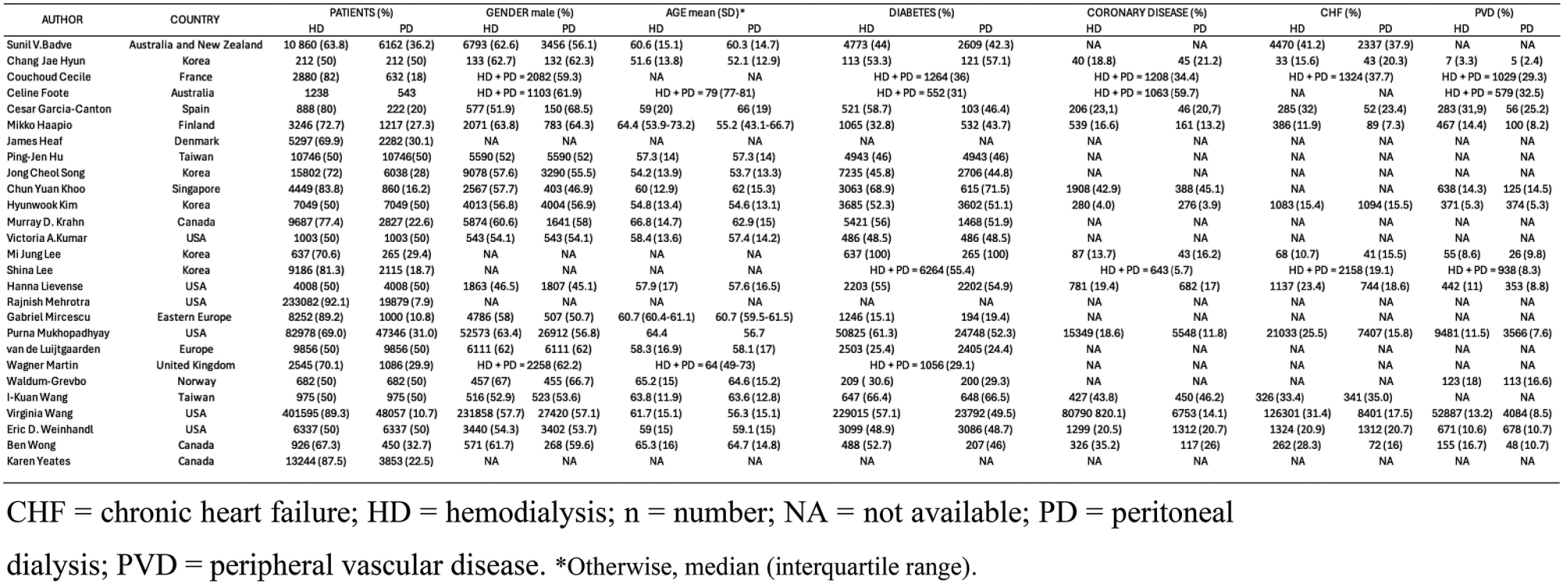
Population characteristics of the included studies

### Risk of bias and study quality

Three studies were considered at high risk of bias primarily due to lack of adjustment for potential confounding variables [37, 49, 53], while four studies were judged to have unclear risk of bias since the modality of the intention to treat analysis was not explained in detail [38, 39, 45, 57] (Supplementary Table S1). On the other hand, the remaining 20 studies were deemed at low risk of bias [13–15, 40–44, 46–48, 50–52, 55, 56, 58–60, 62].

### Overall mortality

**Fig. 2:**
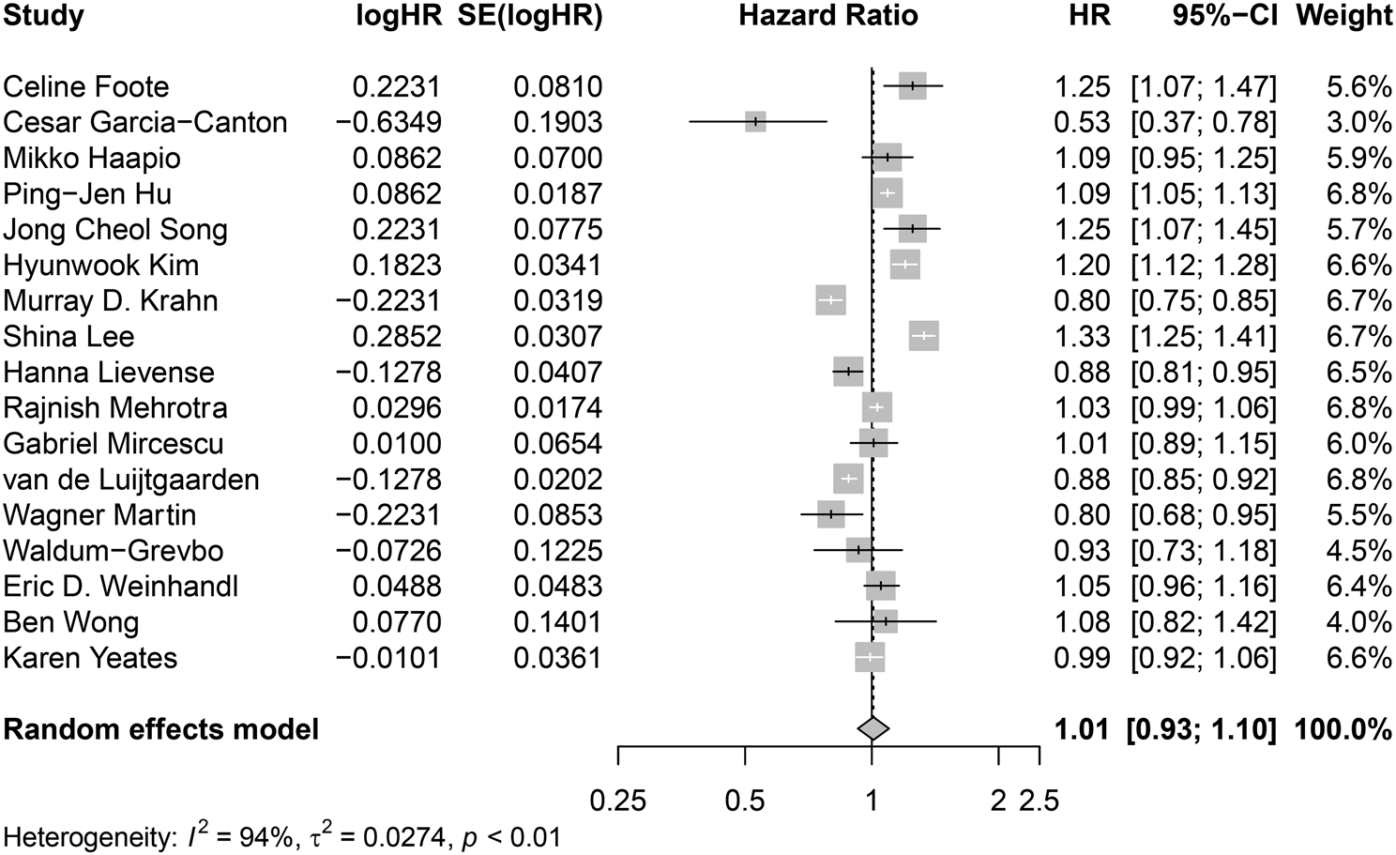
Forest plot for meta-analysis of studies comparing all-cause mortality risk of PD versus in-centre HD. CI = confidence interval; HR = hazard ratio; SE = standard error

Analysis of all the studies showed no statistically significant difference in mortality between PD and incenter HD (HR = 1.06 [95% CI 0.97–1.15, *I*^*2*^ = 94%]), (Fig. 2). There was a significant interaction based on risk of bias, hence, we considered the estimate derived from the 17 studies at low risk of bias to be the best available estimate (HR = 1.01 [95% CI, 0.93–1.10, *I*^*2*^ = 94%]. Subsequent subgroup analyses were performed only on studies at low risk of bias.

### Subgroup analyses

When applying an age cut-off of 65 years, we identified a statistically significant subgroup effect (*p* = 0.01), indicating that age acted as a significant effect modifier and may partially account for the observed heterogeneity. Specifically, in patients younger than 65 years, PD was associated with a significantly lower mortality compared with HD (HR 0.75, 95% CI 0.56–0.99).

There was substantial heterogeneity between the studies within each of these subgroups (age > 65: *I*^*2*^ = 96%; age < 65: *I*^*2*^ = 46%].

**Fig. 3:**
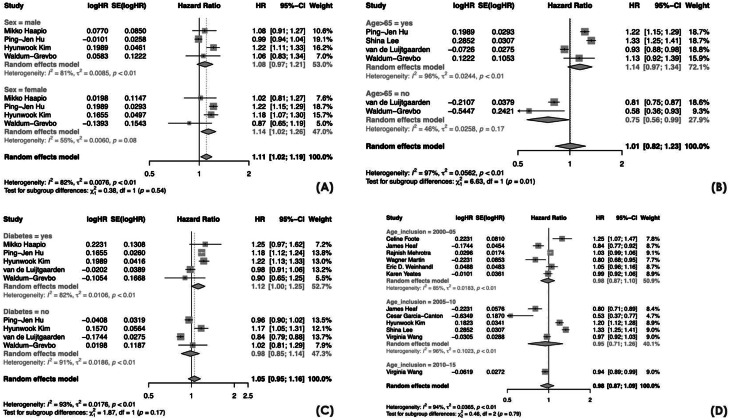
Forest plots for meta-analyses of PD versus HD mortality risk by (**A**) sex; (**B**) age; (**C**) diabetes; (**D**) time period. CI = confidence interval; HR = hazard ratio; SE = standard error

**Fig. 4:**
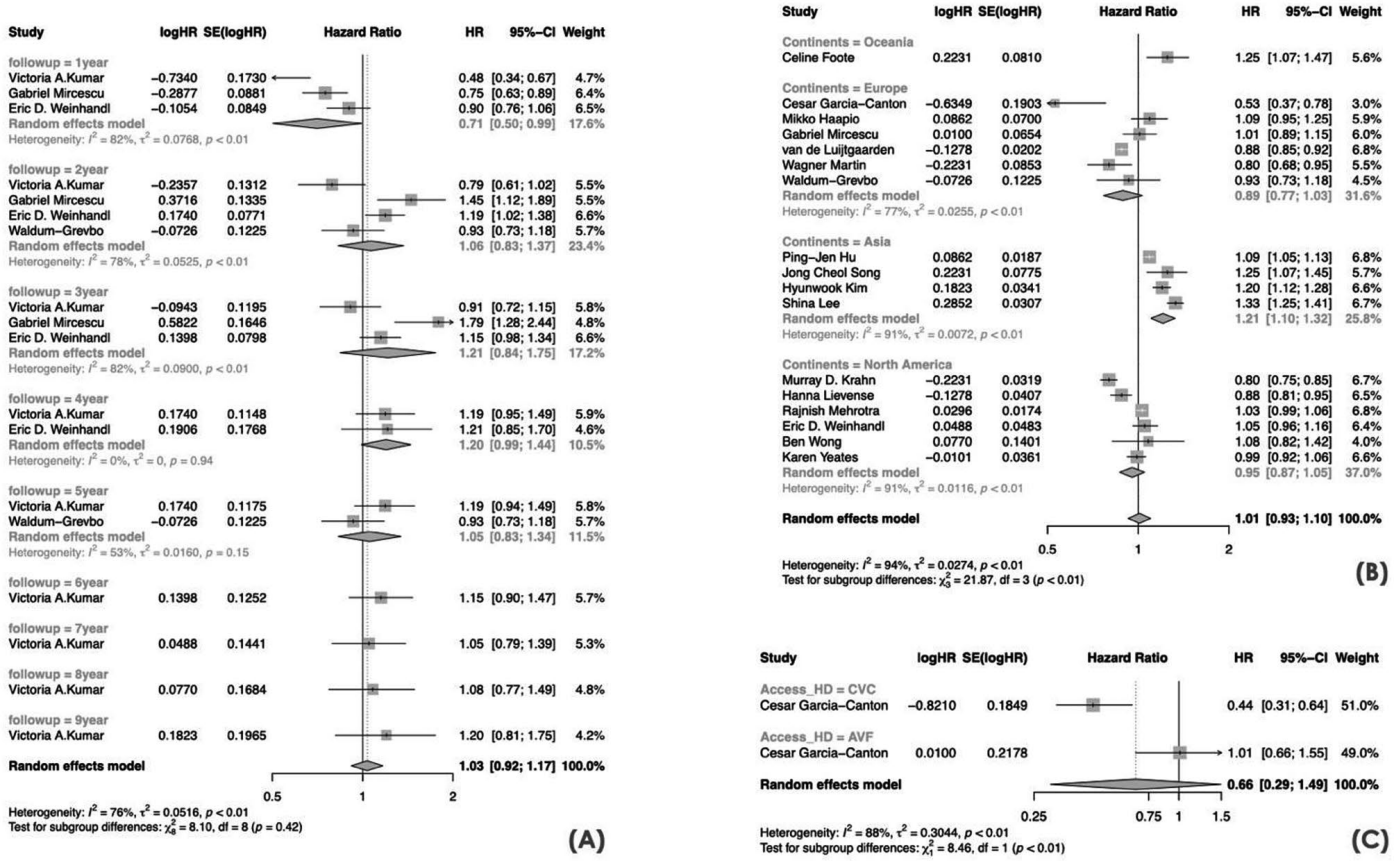
Forest plots for meta-analyses of PD versus HD mortality risk by (**A**) dialysis vintage; (**B**) geographical continent; (**C**) vascular access. CI = confidence interval; HR = hazard ratio; SE = standard error

We also observed a statistically significant subgroup effect when considering the continent of origin of the included studies (Oceania vs. Europe vs. Asia vs. North America; *p* < 0.01), indicating that geographic location was a significant effect modifier. The lowest HRs were observed in Europe (HR 0.89, 95% CI 0.77–1.03) and North America (HR 0.95, 95% CI 0.87–1.05), suggesting comparable or slightly better survival with PD, whereas higher HRs were reported in Oceania (HR 1.25, 95% CI 1.07–1.47) and Asia (HR 1.21, 95% CI 1.10–1.32), indicating higher mortality with PD in those regions. We detected substantial heterogeneity in each subgroup, except Oceania whose result was based on a single study. There was a statistically significant subgroup effect for the vascular access types in HD (*p* < 0.01), but this estimate was derived from only one study. No statistically significant subgroup effect was found for sex, diabetes, dialysis vintage, and study cohort inclusion period (Figs. 3 and 4).

### Certainty of evidence

The GRADE evidence profile is shown in Table 3. Although the presence of heterogeneity among the studies was adequately explained by different baseline features of the included populations, the final certainty of evidence concerning the primary outcome was graded as low, due to the nonrandomized nature of the available studies.

**Table 3:**
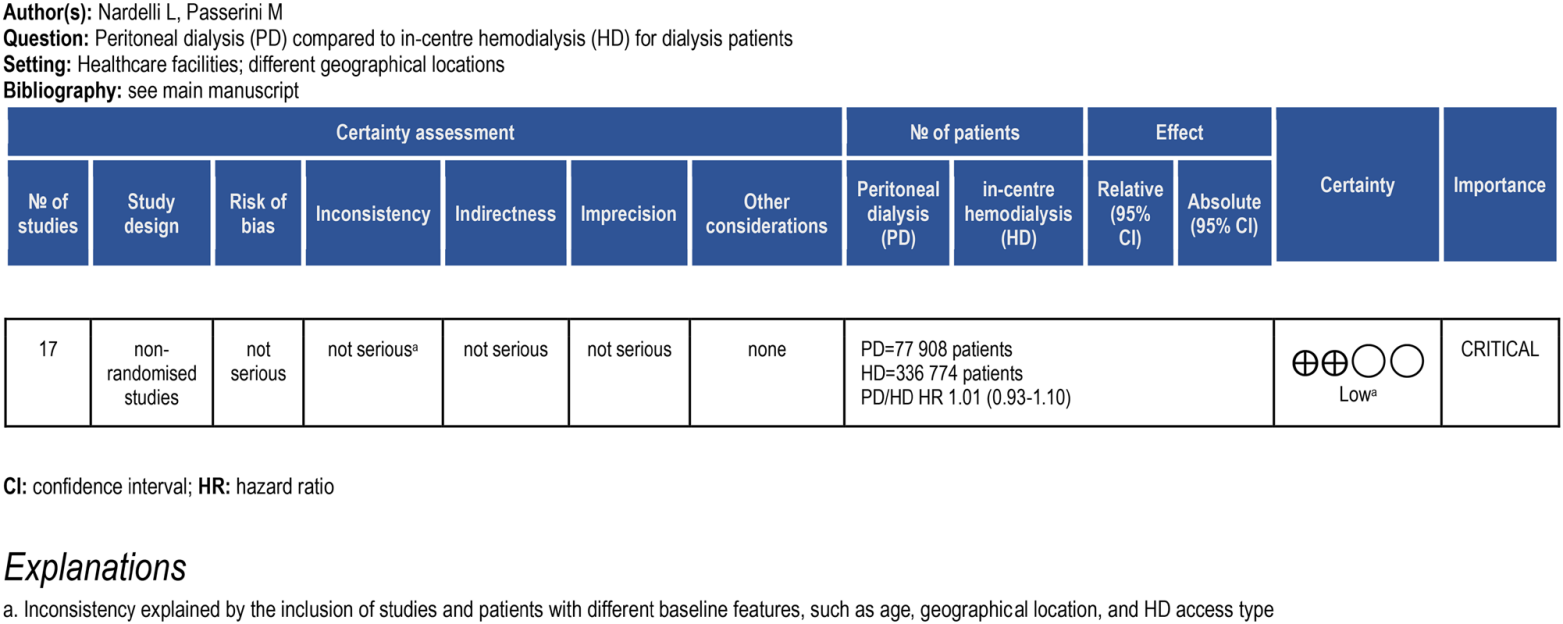
GRADE evidence profile of the primary outcome (overall mortality PD vs. HD patients)

## Discussion

To the best of our knowledge, this is the first systematic review and meta-analysis to comprehensively investigate the survival benefits of PD compared with in-centre HD based exclusively on contemporary cohorts.

In a context where patients randomization to the two different dialysis modalities proved to be extremely challenging (if not unrealistic) [16, 17], previous comparative analysis and systematic reviews of PD/HD mortality were unequivocally limited due to the inclusion of both historical and heterogeneous studies [18–23].

To minimize the effect of selection bias and, at the same time, obtain results which could be reasonably applied to the modern dialytic cohorts, we employed strict inclusion criteria during the process of study selection.

Firstly, we decided to exclude all the data concerning patients who started dialysis before 2000. This year was used as “watershed” since it may correspond to the widespread diffusion of APD [63] and the use of icodextrin in PD practice [64, 65] along with the advent of biocompatible membranes and the dissemination of on-line high flux hemodiafiltration [29, 30]. In addition, to exclude patients at risk for early mortality we considered only studies that classified patients according to the dialysis modality received 90 days post-initiation of therapy [66, 67]. Furthermore, we selected exclusively “intention-to treat” analysis excluding “as-treated” evaluation. “Intention-to treat” analysis allocates patient death based on the starting treatment modality, while “as-treated” analysis allocates patient death according to the treatment modality at the moment of the event. Considering that the rate of transfer of PD patients to HD is significantly higher than the opposite (HD to PD) and that most modality changes are associated with increased mortality after transfers [68, 69], we believed that only analysis adjusting for these changes allows to evaluate the effect of initial treatment modality [70, 71]. Despite all these precautions, the level of certainty of the meta-analysis results according to the GRADE profile [34] was unavoidably categorized as “low”, due to the impossibility to include randomized clinical trials.

In our systematic review and meta-analysis we did not find a significant difference in overall mortality risk between PD and in-center HD patients.

However, the HR of death for PD versus in-center HD was heterogenous among cohort studies [*I*^*2*^ = 94%, *P* < 0.01]. Hence, subgroup analyses were conducted to explore potential sources of heterogeneity, including sex, age, diabetes, dialysis vintage, geographical distribution, HD access, and study cohort inclusion period. The analyses revealed a statistically significant subgroup effect for age, geographical distribution of the individual studies, and HD vascular access. Despite the need for cautious interpretation of subgroup analyses exploring heterogeneity, some issues warrant consideration.

We observed a significant survival benefit for PD in subjects with an age lower than 65 years. These data strengthened the previous evidence that in young patients PD has a survival advantage over HD^22^. Since PD is a self-management treatment and intraluminal peritonitis is the most frequent and impactful event [72, 73], a younger age might help maximize therapy compliance, while minimizing incidence and severity of complications through either a scrupulous execution of the exchanges or/and an early referral of potential hurdles.

Our finding that PD was not associated with inferior survival among diabetic patients contrasts with earlier meta-analyses [21, 22]. For example, Xue et al. reviewed 17 cohort studies and reported that PD was associated with increased mortality compared with HD in diabetic ESRD patients (HR 1.23; 95% CI 1.13–1.34). Similarly, the meta-analysis by Elsayed et al., which included 113,578 propensity score–matched incident dialysis patients from cohorts spanning 1993–2014, found a modestly higher mortality risk for PD versus HD in diabetic patients (HR 1.09; 95% CI 0.98–1.21), although this difference did not consistently reach statistical significance. Additional studies focusing on older populations also suggested a survival disadvantage for PD in diabetic patients, with higher mortality risks [19, 20, 74].

Those outcomes have been traditionally referred to the constant exposure to glucose in the dialysate that may worsen glycemic homeostasis [75]. Furthermore, individuals with diabetes possess more often a higher peritoneal transport rate that could make more challenging the achievement of volume and adequacy targets [76]. However, the advent of new glucose-sparing dialysates [77], such as icodextrins [78] or amino acids [79], along with the routinary use of APD in clinical practice, could explain these different results. Additional factors may justify this discrepancy. First, prior analyses often included older cohorts, when PD solutions, catheter technologies, and infection-prevention strategies were less advanced, resulting in higher technique failure and mortality. In contrast, our study incorporates more contemporary cohorts, reflecting improvements in PD training, solutions, and multidisciplinary support that have particularly benefited higher-risk populations such as patients with diabetes. Second, differences in patient characteristics across studies may contribute; for instance, earlier cohorts included diabetic patients with higher cardiovascular comorbidity burden and poorer glycemic control, both of which strongly influence mortality. Third, regional practice patterns may play a role, as in some healthcare systems PD has historically been offered preferentially to multimorbid patients, whereas in more recent years modality allocation has become more balanced. Currently, the renewed interest for free- or low-glucose peritoneal solutions is demonstrated by ongoing clinical trials which aim at evaluating the efficacy and safety of novel xylitol-carnitine and polydextrine-composed peritoneal solutions [80, 81]. These novelties along with the technological advancements of systems for PD will require soon a re-evaluation of the outcomes regarding individuals with diabetes on PD.

In addition, our analyses challenge the theory that PD offers a survival advantage during the first year of therapy compared to HD, with this benefit diminishing as dialysis vintage increases [13, 48, 52, 57]. Based on this hypothesis, a “PD-first policy” followed by a planned transition to HD has been proposed [82]. Conversely, the notion of PD as the most suitable bridge to kidney transplantation for potential candidates has been reinforced [83].

Our new data may not fully support the applicability of these concepts to modern cohorts and may call for a more cautious approach when considering transition from PD to HD after the first few years of dialysis. Furthermore, in line with evidence from the last two decades that survival among PD has been superior compared to HD [13, 15, 31, 54, 60, 84, 85], we did not detect a statistically significant subgroup effect for study cohort inclusion period.

As mentioned above, with the emergence of new dialysates and dialyzers, improvement of the ultrafiltration capacity, increased attention to preserving residual renal function and peritoneal membrane integrity, improved dialysis adequacy as well as management of anemia, mineral and nutrition parameters, great advances in PD have been accomplished. These advancements, along with the early evidence, call for future analyses in the coming years focused exclusively on data from the last decade.

We detected a statistically significant subgroup effect when considering the continent of origin of the included studies (Oceania vs. Europe vs. Asia vs. North America). Lowest hazard ratios for mortality comparing PD to HD were observed in Europe (HR 0.89, 95% CI 0.77–1.03) and North America (HR 0.95, 95% CI 0.87–1.05), suggesting similar or slightly better survival with PD in these regions. By contrast, higher HRs were obtained in Oceania (HR 1.25, 95% CI 1.07–1.47) and Asia (HR 1.21, 95% CI 1.10–1.32), indicating that in these regions patients on PD had a significantly higher risk of mortality compared with those on HD—thus reflecting a survival advantage for HD.

Peritoneal dialysis technological advancements and solutions are not yet equally accessible worldwide. This, together with differences in the quality of dialysis delivery and patient population characteristics across continents, may contribute to the geographical disparities observed in our analysis. In particular, the higher mortality associated with PD in Asia and Oceania may likely reflect a complex interplay of systemic and clinical factors rather than an inherent inferiority of the modality. Disparities in healthcare infrastructure, including timely access to hospitalization, vascular access services, and multidisciplinary dialysis teams, may disproportionately impact PD patients who depend on rapid intervention for complications such as peritonitis or fluid overload. Variability in patient and caregiver training, home visit programs, and technical support may also influence outcomes, as inadequate training has consistently been linked to higher rates of technique failure. Moreover, differences in registry coverage, data completeness, and outcome definitions could introduce reporting bias and contribute to heterogeneity in results. Finally, modality allocation policies differ across regions: in some Asian countries, PD is more often offered to socioeconomically disadvantaged or higher-risk patients due to cost constraints or policy considerations, whereas in Europe and North America, modality choice tends to be more patient-driven.

Unfortunately, it was not possible to extensively investigate by meta-analysis the effect of HD vascular access types on the relationship of dialysis modality and mortality. The only available study confirms that PD has a clear survival benefit over HD patients starting dialysis with a CVC [41], reinforcing the potentially advantage that PD could confer to subjects who are judged ineligible for arteriovenous fistula creation [67]. Conversely, new data are required to explore the mortality outcome among incident PD patients and subjects initiating HD with an AVF.

Despite the rigorous methodology employed in this systematic review and meta-analysis, several limitations should be acknowledged. First, as the evidence is derived entirely from observational studies, it is inherently less robust than data from randomized controlled trials. Second, although most included studies adjusted for known confounders, residual confounding from unknown or unmeasured variables may still affect the validity of the findings. Third, the potential for immortal time bias remains, despite the classification of patients according to dialysis modality at 90 days post-initiation, as this approach cannot completely eliminate residual bias in the absence of individual patient data. Fourth, many studies were registry-based, which introduces the possibility of coding inaccuracies, incomplete records, and population overlap. Fifth, substantial heterogeneity was observed in the main analysis, and while predefined subgroup analyses were conducted, meta-regression was not performed due to uneven distribution of covariates across studies; nonetheless, exclusion of potential outliers did not materially alter the results. Finally, the analysis lacks representation from South America, the Middle East, and Africa due to limited regional registry data, which may limit the generalizability of the findings. These limitations must be considered alongside the strengths of the study, including the exclusion of historical cohorts, the use of clearly defined inclusion criteria, and a systematic approach to minimize bias.

In the future, target trial emulation—by explicitly defining the protocol of a hypothetical randomized controlled trial and emulating it using observational data—could enhance transparency, mitigate biases such as immortal time and selection bias, and allow adjustment for key covariates, including healthcare system characteristics, geographical location, diabetic status, residual kidney function at dialysis initiation, HD vascular access type, exposure to modern PD solutions, and year of dialysis initiation. Leveraging large registries and electronic health records, this methodology has the potential to strengthen causal inference and generate more reliable evidence to inform clinical practice [86]. In conclusion, evidence derived from the results of this systematic review and meta-analysis evaluating the effect of incident dialysis modality on mortality indicates that, overall, PD and in-center HD carry similar survival rates. However, differences were detected among subgroups. These discrepancies seem to depend mainly on age and continent of origin, but not on sex, diabetic status, and dialysis vintage.

Given the significant challenges and impracticality of conducting randomized controlled trials in this setting, this study offers valuable reassurance that PD and in-center HD provide equivalent overall survival in contemporary cohorts. As such, dialysis modality selection should be individualized, considering patient preferences, clinical characteristics, lifestyle, and access to care. PD may confer a survival advantage in patients under 65 years of age and remains a viable option for individuals with diabetes—challenging earlier assumptions of inferior outcomes in this population. Additionally, PD should be strongly considered for patients without arteriovenous fistula access, given the higher mortality associated with initiating HD via central venous catheter. The findings also challenge the traditional “PD-first, then HD” strategy, as no decline in survival over time was observed among patients maintained on PD. When well-tolerated and clinically effective, PD can be safely continued as a long-term treatment. These results support broader utilization of PD in appropriate patients and reinforce the importance of shared decision-making in selecting dialysis modality.

## Supplementary Information

Below is the link to the electronic supplementary material.

Supplementary Material 1

## Data Availability

the datasets used and/or analyzed during the current study are available from the corresponding author on reasonable request.
