# Supplementary material for: Association of incident dialysis modality with patient survival: a systematic review and meta-analysis
Source: BMC Nephrol. 2025 Oct 24;26:588. doi: 10.1186/s12882-025-04530-4 (PMC12553293; doi:10.1186/s12882-025-04530-4)
Supplement: Supplementary file 1 — Supplementary Material 1 [file 12882_2025_4530_MOESM1_ESM.docx]

**SUPPLEMENTARY**

**Actual search strategies………………………………………………………………..pag.2**

**Risk of bias assessment results………………………………………………………pag.7**

**PRISMA 2020 main checklist…………………………………………………………..pag.8**

**PRISMA abstract checklist……………………………………………………………..pag.12**

**ACTUAL SEARCH STRATEGIES**

**OVID**

Database(s): **Embase**1988 to 2022 Week 15**, Ovid MEDLINE(R) and Epub Ahead of Print, In-Process, In-Data-Review & Other Non-Indexed Citations**1996 to April 21, 2022**, EBM Reviews - Cochrane Central Register of Controlled Trials**March 2022**, EBM Reviews - Cochrane Database of Systematic Reviews**2005 to April 20, 2022
Search Strategy:

| **#** | **Searches** |
| --- | --- |
| 1 | Hemodiafiltration/ or *hemodialysis/ |
| 2 | (hemodialysis or haemodialysis or hemofiltrat* or haemofiltrat* or hemodiafiltrat* or haemodiafiltrat* or "acid-free biofiltration" or ((renal or kidney) adj1 dialysis)).ti,ab,hw,kw,kf. |
| 3 | 1 or 2 |
| 4 | exp Peritoneal Dialysis/ |
| 5 | ((peritoneal or peritoneum) adj3 dialysis).ti,ab,hw,kw,kf. |
| 6 | 4 or 5 |
| 7 | 3 and 6 |
| 8 | exp Survival/ or Survival Rate/ or exp survival analysis/ |
| 9 | exp Mortality/ |
| 10 | exp Death/ |
| 11 | Life Expectancy/ |
| 12 | (surviv* or death or mortality).ti,ab,hw,kw,kf. |
| 13 | mt.fs. |
| 14 | or/8-13 |
| 15 | (modalit* or method or methods or technique* or efficacy or efficacious or effective*or versus or choice or choose or chose or chosen or compar*).ti,ab,hw,kw,kf. |
| 16 | 7 and 14 and 15 |
| 17 | (modalit* adj7 (dialysis or "renal replacement")).ti. |
| 18 | 14 and 17 |
| 19 | 16 or 18 |
| 20 | (conference abstract or conference review or editorial or erratum or note or addresses or autobiography or bibliography or biography or blogs or comment or dictionary or directory or interactive tutorial or interview or lectures or legal cases or legislation or news or newspaper article or patient education handout or periodical index or portraits or published erratum or video-audio media or webcasts).mp. or conference abstract.st. |
| 21 | 19 not 20 |
| 22 | (("animal stud*" or "animal research" or alpaca or alpacas or algae* or amphibian or amphibians or animal or animals or antelope or armadillo or armadillos or avian or baboon or baboons or bats or beagle or beagles or bee or bees or bird or birds or bison or bovine or buffalo or buffaloes or buffalos or "c elegans" or "Caenorhabditis elegans" or camel or camels or canine or canines or canis or carp or cats or catfish or cattle or chamaeleo* or chameleon* or chick or chicken or chickens or chicks or chimp or chimpanze or chimpanzees or chimps or cow or cows or "D melanogaster" or "dairy calf" or "dairy calves" or deer or dog or dogs or donkey or donkeys or drosophila or "Drosophila melanogaster" or duck or duckling or ducklings or ducks or equid or equids or equine or equines or feline or felines or ferret or ferrets or finch or finches or fish or flatworm or flatworms or fox or foxes or frog or frogs or "fruit flies" or "fruit fly" or "G mellonella" or "Galleria mellonella" or geese or gerbil or gerbils or goat or goats or goose or gorilla or gorillas or groundhog or groundhogs or hamster or hamsters or hare or hares or heifer or heifers or horse or horses or iguana or iguanas or insect or insects or jellyfish or kangaroo or kangaroos or kitten or kittens or "laboratory animal*" or lagomorph or lagomorphs or lamb or lambs or lemur or lemurs or lemuridae or llama or llamas or macaque or macaques or macaw or macaws or marmoset or marmosets or mice or minipig or minipigs or mink or minks or monkey or monkeys or mouse or mule or mules or muskrat or muskrats or nematode or nematodes or newt or newts or octopus or octopuses or orangutan or "orang-utan" or orangutans or "orang-utans" or oxen or parrot or parrots or pig or pigeon or pigeons or piglet or piglets or pigs or porcine or primate or primates or poultry or quail or rabbit or rabbits or rat or rats or reptile or reptiles or rodent or rodents or ruminant or ruminants or salmon or sheep or shrimp or slug or slugs or swine or tamarin or tamarins or tilapia or tilapias or toad or toads or trout or urchin or urchins or vole or voles or waxworm or waxworms or weasel or weasels or wolf or wolves or worm or worms or wrass* or xenopus or "zebra fish" or zebrafish) not (human or humans or patient or patients)).ti,ab,hw,kw. |
| 23 | (rat or rats or mice or mouse or murine or pig or pigs or porcine or swine or dog or dogs).ti. |
| 24 | 22 or 23 |
| 25 | 21 not 24 |
| 26 | limit 25 to yr="2000 -Current" |
| 27 | remove duplicates from 26 |

**SCOPUS via Elsevier 1788 +**

| 1 | ( TITLE-ABS-KEY ( hemodialysis OR haemodialysis OR hemofiltrat* OR haemofiltrat* OR hemodiafiltrat* OR haemodiafiltrat* OR "acid-free biofiltration" OR ( ( renal OR kidney ) W/ dialysis )) AND TITLE-ABS-KEY ( ( peritoneal OR peritoneum ) W/3 dialysis ) ) AND ( TITLE-ABS-KEY ( surviv* OR death OR mortality ) ) |
| --- | --- |
| 2 | INDEX(embase) OR INDEX(medline) OR PMID(0* OR 1* OR 2* OR 3* OR 4* OR 5* OR 6* OR 7* OR 8* OR 9*) |
| 3 | 1 not 2 |
| 4 | PUBYEAR AFT 1999 |
| 5 | 3 and 4 |
| 6 | ( TITLE-ABS-KEY ( ( alpaca OR alpacas OR amphibian OR amphibians OR animal OR animals OR antelope OR armadillo OR armadillos OR avian OR baboon OR baboons OR beagle OR beagles OR bee OR bees OR bird OR birds OR bison OR bovine OR buffalo OR buffaloes OR buffalos OR "c elegans" OR "Caenorhabditis elegans" OR camel OR camels OR canine OR canines OR carp OR cats OR cattle OR chick OR chicken OR chickens OR chicks OR chimp OR chimpanze OR chimpanzees OR chimps OR cow OR cows OR "D melanogaster" OR "dairy calf" OR "dairy calves" OR deer OR dog OR dogs OR donkey OR donkeys OR drosophila OR "Drosophila melanogaster" OR duck OR duckling OR ducklings OR ducks OR equid OR equids OR equine OR equines OR feline OR felines OR ferret OR ferrets OR finch OR finches OR fish OR flatworm OR flatworms OR fox OR foxes OR frog OR frogs OR "fruit flies" OR "fruit fly" OR "G mellonella" OR "Galleria mellonella" OR geese OR gerbil OR gerbils OR goat OR goats OR goose OR gorilla OR gorillas OR hamster OR hamsters OR hare OR hares OR heifer OR heifers OR horse OR horses OR insect OR insects OR jellyfish OR kangaroo OR kangaroos OR kitten OR kittens OR lagomorph OR lagomorphs OR lamb OR lambs OR llama OR llamas OR macaque OR macaques OR macaw OR macaws OR marmoset OR marmosets OR mice OR minipig OR minipigs OR mink OR minks OR monkey OR monkeys OR mouse OR mule OR mules OR nematode OR nematodes OR octopus OR octopuses OR orangutan OR "orang-utan" OR orangutans OR "orang-utans" OR oxen OR parrot OR parrots OR pig OR pigeon OR pigeons OR piglet OR piglets OR pigs OR porcine OR primate OR primates OR quail OR rabbit OR rabbits OR rat OR rats OR reptile OR reptiles OR rodent OR rodents OR ruminant OR ruminants OR salmon OR sheep OR shrimp OR slug OR slugs OR swine OR tamarin OR tamarins OR toad OR toads OR trout OR urchin OR urchins OR vole OR voles OR waxworm OR waxworms OR worm OR worms OR xenopus OR "zebra fish" OR zebrafish ) AND NOT ( human OR humans OR patient OR patients ) ) ) |
| 7 | 5 not 6 |
| 8 | DOCTYPE(ed) OR DOCTYPE(bk) OR DOCTYPE(er) OR DOCTYPE(no) OR DOCTYPE(sh) OR DOCTYPE(ch) |
| 9 | 7 not 8 |

**Web of Science Core Collection via Clarivate Analytics (1975+)**

| 1 | (TS=(hemodialysis OR haemodialysis OR hemofiltrat* OR haemofiltrat* OR hemodiafiltrat* OR haemodiafiltrat* OR "acid-free biofiltration" OR ((renal or kidney) NEAR dialysis)) AND TS=((peritoneal OR peritoneum) NEAR/3 dialysis)) AND (TS=(surviv* OR death OR mortality)) |
| --- | --- |
| 2 | PMID=(0* or 1* or 2* or 3* or 4* or 5* or 6* or 7* or 8* or 9*) |
| 3 | 1 not 2 |
| 4 | 3 and Timespan: 2000 to 2022 |
| 5 | TS=((alpaca OR alpacas OR amphibian OR amphibians OR animal OR animals OR antelope OR armadillo OR armadillos OR avian OR baboon OR baboons OR beagle OR beagles OR bee OR bees OR bird OR birds OR bison OR bovine OR buffalo OR buffaloes OR buffalos OR "c elegans" OR "Caenorhabditis elegans" OR camel OR camels OR canine OR canines OR carp OR cats OR cattle OR chick OR chicken OR chickens OR chicks OR chimp OR chimpanze OR chimpanzees OR chimps OR cow OR cows OR "D melanogaster" OR "dairy calf" OR "dairy calves" OR deer OR dog OR dogs OR donkey OR donkeys OR drosophila OR "Drosophila melanogaster" OR duck OR duckling OR ducklings OR ducks OR equid OR equids OR equine OR equines OR feline OR felines OR ferret OR ferrets OR finch OR finches OR fish OR flatworm OR flatworms OR fox OR foxes OR frog OR frogs OR "fruit flies" OR "fruit fly" OR "G mellonella" OR "Galleria mellonella" OR geese OR gerbil OR gerbils OR goat OR goats OR goose OR gorilla OR gorillas OR hamster OR hamsters OR hare OR hares OR heifer OR heifers OR horse OR horses OR insect OR insects OR jellyfish OR kangaroo OR kangaroos OR kitten OR kittens OR lagomorph OR lagomorphs OR lamb OR lambs OR llama OR llamas OR macaque OR macaques OR macaw OR macaws OR marmoset OR marmosets OR mice OR minipig OR minipigs OR mink OR minks OR monkey OR monkeys OR mouse OR mule OR mules OR nematode OR nematodes OR octopus OR octopuses OR orangutan OR "orangutan" OR orangutans OR "orang-utans" OR oxen OR parrot OR parrots OR pig OR pigeon OR pigeons OR piglet OR piglets OR pigs OR porcine OR primate OR primates OR quail OR rabbit OR rabbits OR rat OR rats OR reptile OR reptiles OR rodent OR rodents OR ruminant OR ruminants OR salmon OR sheep OR shrimp OR slug OR slugs OR swine OR tamarin OR tamarins OR toad OR  toads OR trout OR urchin OR urchins OR vole OR voles OR waxworm OR waxworms OR worm OR worms OR xenopus OR "zebra fish" OR zebrafish) NOT (human OR humans)) |
| 6 | 4 not 5 |

**RISK OF BIAS ASSESSMENT RESULTS**


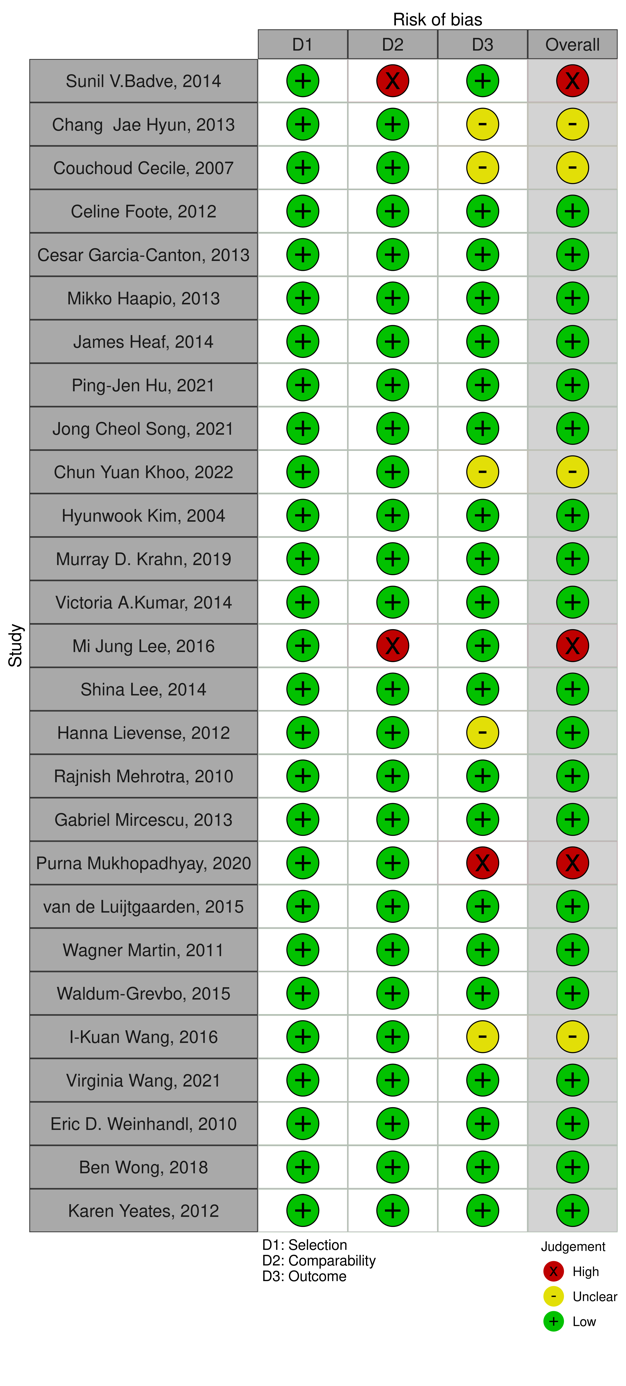


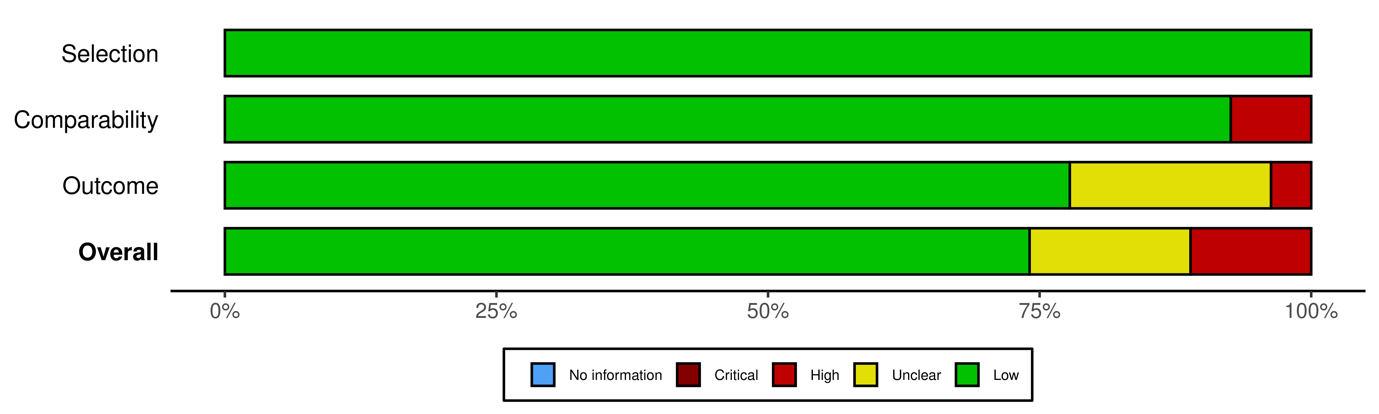


**Table S1 Risk of Bias Assessments**

*From:* McGuinness, LA, Higgins, JPT. Risk-of-bias VISualization (robvis): An R package and Shiny web app for visualizing risk-of-bias assessments. Res Syn Meth. 2020; 1- 7. https://doi.org/10.1002/jrsm.1411

# **PRISMA 2020 MAIN CHECKLIST**

| **Topic** | **No.** | **Item** | **Location where item is reported** |
| --- | --- | --- | --- |
| **TITLE** |  |  |  |
| **Title** | 1 | Identify the report as a systematic review. | Title |
| **ABSTRACT** |  |  |  |
| **Abstract** | 2 | See the PRISMA 2020 for Abstracts checklist |  |
| **INTRODUCTION** |  |  |  |
| **Rationale** | 3 | Describe the rationale for the review in the context of existing knowledge. | lines 81-85 |
| **Objectives** | 4 | Provide an explicit statement of the objective(s) or question(s) the review addresses. | lines 97-98 |
| **METHODS** |  |  |  |
| **Eligibility criteria** | 5 | Specify the inclusion and exclusion criteria for the review and how studies were grouped for the syntheses. | lines 121-135 |
| **Information sources** | 6 | Specify all databases, registers, websites, organisations, reference lists and other sources searched or consulted to identify studies. Specify the date when each source was last searched or consulted. | lines 103-115; Supplementary |
| **Search strategy** | 7 | Present the full search strategies for all databases, registers and websites, including any filters and limits used. | Supplementary |
| **Selection process** | 8 | Specify the methods used to decide whether a study met the inclusion criteria of the review, including how many reviewers screened each record and each report retrieved, whether they worked independently, and if applicable, details of automation tools used in the process. | lines 136-138 |
| **Data collection process** | 9 | Specify the methods used to collect data from reports, including how many reviewers collected data from each report, whether they worked independently, any processes for obtaining or confirming data from study investigators, and if applicable, details of automation tools used in the process. | lines 138-142 |
| **Data items** | 10a | List and define all outcomes for which data were sought. Specify whether all results that were compatible with each outcome domain in each study were sought (e.g. for all measures, time points, analyses), and if not, the methods used to decide which results to collect. | lines 150-154 |
|  | 10b | List and define all other variables for which data were sought (e.g. participant and intervention characteristics, funding sources). Describe any assumptions made about any missing or unclear information. | lines 154-157 |
| **Study risk of bias assessment** | 11 | Specify the methods used to assess risk of bias in the included studies, including details of the tool(s) used, how many reviewers assessed each study and whether they worked independently, and if applicable, details of automation tools used in the process. | lines 145-146 |
| **Effect measures** | 12 | Specify for each outcome the effect measure(s) (e.g. risk ratio, mean difference) used in the synthesis or presentation of results. | lines 150-151 |
| **Synthesis methods** | 13a | Describe the processes used to decide which studies were eligible for each synthesis (e.g. tabulating the study intervention characteristics and comparing against the planned groups for each synthesis (item 5)). | lines 121-135 |
|  | 13b | Describe any methods required to prepare the data for presentation or synthesis, such as handling of missing summary statistics, or data conversions. | n/a |
|  | 13c | Describe any methods used to tabulate or visually display results of individual studies and syntheses. | Table 1; Table 2 |
|  | 13d | Describe any methods used to synthesize results and provide a rationale for the choice(s). If meta-analysis was performed, describe the model(s), method(s) to identify the presence and extent of statistical heterogeneity, and software package(s) used. | lines 151-154 |
|  | 13e | Describe any methods used to explore possible causes of heterogeneity among study results (e.g. subgroup analysis, meta-regression). | lines 154-158 |
|  | 13f | Describe any sensitivity analyses conducted to assess robustness of the synthesized results. | n/a |
| **Reporting bias assessment** | 14 | Describe any methods used to assess risk of bias due to missing results in a synthesis (arising from reporting biases). | n/a |
| **Certainty assessment** | 15 | Describe any methods used to assess certainty (or confidence) in the body of evidence for an outcome. | lines 146-147 |
| **RESULTS** |  |  |  |
| **Study selection** | 16a | Describe the results of the search and selection process, from the number of records identified in the search to the number of studies included in the review, ideally using a flow diagram. | lines 165-169; Figure 1 |
|  | 16b | Cite studies that might appear to meet the inclusion criteria, but which were excluded, and explain why they were excluded. | Figure 1 |
| **Study characteristics** | 17 | Cite each included study and present its characteristics. | Table 1; line 169 |
| **Risk of bias in studies** | 18 | Present assessments of risk of bias for each included study. | Supplementary |
| **Results of individual studies** | 19 | For all outcomes, present, for each study: (a) summary statistics for each group (where appropriate) and (b) an effect estimate and its precision (e.g. confidence/credible interval), ideally using structured tables or plots. | Table 2 |
| **Results of syntheses** | 20a | For each synthesis, briefly summarise the characteristics and risk of bias among contributing studies. | Table 1; Supplementary |
|  | 20b | Present results of all statistical syntheses conducted. If meta-analysis was done, present for each the summary estimate and its precision (e.g. confidence/credible interval) and measures of statistical heterogeneity. If comparing groups, describe the direction of the effect. | lines 192-196 |
|  | 20c | Present results of all investigations of possible causes of heterogeneity among study results. | lines 200-216 |
|  | 20d | Present results of all sensitivity analyses conducted to assess the robustness of the synthesized results. | n/a |
| **Reporting biases** | 21 | Present assessments of risk of bias due to missing results (arising from reporting biases) for each synthesis assessed. | n/a |
| **Certainty of evidence** | 22 | Present assessments of certainty (or confidence) in the body of evidence for each outcome assessed. | Table 3 |
| **DISCUSSION** |  |  |  |
| **Discussion** | 23a | Provide a general interpretation of the results in the context of other evidence. | lines 265-331 |
|  | 23b | Discuss any limitations of the evidence included in the review. | lines 338-353 |
|  | 23c | Discuss any limitations of the review processes used. | lines 339-351 |
|  | 23d | Discuss implications of the results for practice, policy, and future research. | lines 354-377 |
| **OTHER INFORMATION** |  |  |  |
| **Registration and protocol** | 24a | Provide registration information for the review, including register name and registration number, or state that the review was not registered. | lines 116-117 |
|  | 24b | Indicate where the review protocol can be accessed, or state that a protocol was not prepared. | lines 116-117 |
|  | 24c | Describe and explain any amendments to information provided at registration or in the protocol. | n/a |
| **Support** | 25 | Describe sources of financial or non-financial support for the review, and the role of the funders or sponsors in the review. | In the dedicated section |
| **Competing interests** | 26 | Declare any competing interests of review authors. | In the dedicated section |
| **Availability of data, code and other materials** | 27 | Report which of the following are publicly available and where they can be found: template data collection forms; data extracted from included studies; data used for all analyses; analytic code; any other materials used in the review. | In the dedicated section |

#####

#

# **PRISMA ABSTRACT CHECKLIST**

| **Topic** | **No.** | **Item** | **Reported?** |
| --- | --- | --- | --- |
| **TITLE** |  |  |  |
| **Title** | 1 | Identify the report as a systematic review. | Yes |
| **BACKGROUND** |  |  |  |
| **Objectives** | 2 | Provide an explicit statement of the main objective(s) or question(s) the review addresses. | Yes |
| **METHODS** |  |  |  |
| **Eligibility criteria** | 3 | Specify the inclusion and exclusion criteria for the review. | Yes |
| **Information sources** | 4 | Specify the information sources (e.g. databases, registers) used to identify studies and the date when each was last searched. | No |
| **Risk of bias** | 5 | Specify the methods used to assess risk of bias in the included studies. | No |
| **Synthesis of results** | 6 | Specify the methods used to present and synthesize results. | Yes |
| **RESULTS** |  |  |  |
| **Included studies** | 7 | Give the total number of included studies and participants and summarise relevant characteristics of studies. | Yes |
| **Synthesis of results** | 8 | Present results for main outcomes, preferably indicating the number of included studies and participants for each. If meta-analysis was done, report the summary estimate and confidence/credible interval. If comparing groups, indicate the direction of the effect (i.e. which group is favoured). | Yes |
| **DISCUSSION** |  |  |  |
| **Limitations of evidence** | 9 | Provide a brief summary of the limitations of the evidence included in the review (e.g. study risk of bias, inconsistency and imprecision). | No |
| **Interpretation** | 10 | Provide a general interpretation of the results and important implications. | Yes |
| **OTHER** |  |  |  |
| **Funding** | 11 | Specify the primary source of funding for the review. | No |
| **Registration** | 12 | Provide the register name and registration number. | No |

*From:* Page MJ, McKenzie JE, Bossuyt PM, Boutron I, Hoffmann TC, Mulrow CD, et al. The PRISMA 2020 statement: an updated guideline for reporting systematic reviews. MetaArXiv. 2020, September 14. DOI: 10.31222/osf.io/v7gm2. For more information, visit: [www.prisma-statement.org](file:///Users/luca/Desktop/Meta-analysis/SUB/BMC%20neph/www.prisma-statement.org)
